# DEVELOPMENT AND EVALUATION OF EDUCATIONAL MATERIALS REGARDING MENTAL HEALTH MOBILE APPS AMONG OLDER VETERANS

**DOI:** 10.1093/geroni/igz038.1200

**Published:** 2019-11-08

**Authors:** Ashley Scales, Julia Loup, Christine Juang, Erin Sakai, Flora Ma, Christine E Gould

**Affiliations:** 1 VA Palo Alto Health Care System, Palo Alto, California, United States; 2 University of Alabama, Department of Psychology, Tuscaloosa, Alabama, United States; 3 VA Palo Alto, Palo Alto, California, United States; 4 Palo Alto University, Palo Alto, California, United States; 5 VA Palo Alto Health Care System, Palo Alto, United States

## Abstract

The number of older adults using mobile devices has doubled over recent years; however, many need assistance in learning how to use their device. To address this gap, we developed patient education materials teaching older Veterans how to download apps and the basics of mobile device and app use. For example, we developed step-by-step guides for three Veteran Affairs mobile apps that target mental health symptoms. Material development involved feedback from providers and older Veterans using a multi-step mixed methods evaluation process. Local technology and geriatric content experts provided initial feedback; all experts agreed the materials would be helpful to teach Veterans about mental health apps. We subsequently interviewed older Veterans (M = 78.5 years) who evaluated the materials. Over 50% of Veterans found the guides clear, articulate, and useful; 83.3% noted they would recommend to others. Lastly, providers who see older Veterans regularly rated the materials; 79% of providers rated the materials as helpful, with an average rating of 4.3 (1 = Strongly Disagree to 5 = Strongly Agree). Providers viewed the materials and apps as useful supplements to psychotherapy and especially useful for individuals who are unable to return to clinic. Overall, both providers and Veterans found the materials easy to understand and valuable for those new using mobile apps or devices. Findings from the evaluation process suggest the design of the materials may be vital to increasing the use of mental health mobile apps among older Veterans.

